# From Fibrosis to Malignancy: Mechanistic Intersections Driving Lung Cancer Progression

**DOI:** 10.3390/cancers17233861

**Published:** 2025-12-01

**Authors:** Bing Chen, Hayam Hamdy, Xu Zhang, Pengxiu Cao, Yi Fu, Junling Shen

**Affiliations:** 1 School of Medicine, Kunming University, Kunming 650214, China; chenbing@kmu.edu.cn; 2 Yunnan Key Laboratory of Cell Metabolism and Diseases, Center for Life Sciences, School of Life Sciences, Yunnan University, Kunming 650091, China; 20249075@staff.ynu.edu.cn (H.H.); xuzhang@ynu.edu.cn (X.Z.); 3 College of Life Sciences, Hebei Normal University, Shijiazhuang 050025, China; pengxiucao@hebtu.edu.cn; 4 The Third Affiliated Hospital of Yunnan University of Chinese Medicine, Kunming Municipal Hospital of Traditional Chinese Medicine, Kunming 650011, China

**Keywords:** pulmonary fibrosis, lung cancer, extracellular matrix, tumor microenvironment, proliferation

## Abstract

**Background/Objectives:** Pulmonary fibrosis (PF) and lung cancer (LC) are major global health challenges that share several pathogenic mechanisms despite their distinct clinical features. PF leads to progressive fibrotic remodeling and respiratory decline, while LC is characterized by uncontrolled proliferation, invasion, and metastasis. Growing evidence shows that PF markedly increases the risk of LC development. This review aims to clarify the convergent molecular and cellular mechanisms that link fibrogenesis to tumorigenesis. **Methods:** Published studies exploring shared pathogenic pathways, molecular signaling networks, immune microenvironment alterations, and mitochondrial and genomic disturbances in PF and LC were systematically examined and integrated to identify common mechanisms contributing to fibrosis-associated carcinogenesis. **Results:** Findings highlight several overlapping processes between PF and LC, including oxidative stress, genomic instability, dysregulated DNA damage repair, immune microenvironment remodeling, mitochondrial dysfunction, and alterations in the ubiquitin–proteasome system. These aberrations drive chronic inflammation, epithelial–mesenchymal transition (EMT), extracellular matrix (ECM) remodeling, and other hallmarks shared by both diseases. Key signaling pathways—such as transforming growth factor-β (TGF-β), programmed cell death protein-1/programmed death-ligand 1 (PD-1/PD-L1), and tumor microenvironment–mediated immune evasion—further contribute to disease progression and increased LC risk in PF patients. **Conclusions:** Integrating molecular and pathological insights reveals a strong biological continuum between PF and LC. Understanding these convergent mechanisms may facilitate the identification of diagnostic biomarkers and therapeutic targets, ultimately helping to mitigate PF-associated lung carcinogenesis.

## Simple Summary

Lung cancer often develops in patients with pulmonary fibrosis, but the link between the two diseases remains unclear. This review examines how inflammation, oxidative stress, and alterations in molecular signaling in fibrosis may contribute to the development of lung cancer. Understanding these shared mechanisms could help identify new targets for the early detection and treatment of lung cancer in patients with fibrotic lungs.

## 1. Introduction

Pulmonary fibrosis (PF) and lung cancer (LC) are among the most significant causes of morbidity and mortality worldwide. PF, a chronic and progressive interstitial lung disease, represents the end stage of various pulmonary disorders and is characterized by excessive deposition of fibrotic tissue, resulting in irreversible architectural distortion and functional decline. Idiopathic pulmonary fibrosis (IPF), the most prevalent form of PF, remains of unknown etiology but is associated with several risk factors, including genetic susceptibility, cigarette smoking, environmental exposure, microbial infection, gastroesophageal reflux, and metabolic disorders such as diabetes mellitus [1,2]. Epidemiological studies estimate the global incidence and prevalence of IPF to range from 0.09 to 1.30 and 0.33–4.51 cases per 10,000 individuals, respectively, with a rising trend in aging populations [3]. The relationship between PF and LC is critical, given LC’s status as the leading cause of cancer-related deaths worldwide, primarily classified as non-small-cell lung cancer (NSCLC) and small-cell lung cancer (SCLC), with SCLC and NSCLC accounting for approximately 15% and 85% of cases, respectively [4]. IPF patients have an approximately eightfold higher risk of developing LC compared to the general population, with reported prevalence rates ranging from 2.7% to 31.3% [5]. LC remains the leading cause of cancer-related mortality worldwide, encompassing two major histological types: non-small-cell lung cancer (NSCLC, ~85%) and small-cell lung cancer (SCLC, ~15%) [4]. The multifactorial etiology of LC involves tobacco exposure, environmental pollutants, genetic mutations, and socioeconomic determinants, all of which contribute to disease heterogeneity and therapeutic challenges [6,7]. The 2020 Global Cancer Statistics report confirmed lung cancer as one of the leading causes of cancer death, with an estimated 2.2 million fatalities [8]. While the epidemiological association between PF and LC is well established, this review focuses on elucidating the mechanistic interplay between these two diseases. We aim to clarify how shared molecular pathways drive lung carcinogenesis within fibrotic lung tissue and how the fibrotic microenvironment shapes tumor behavior. In support of this objective, findings from recent studies were incorporated to highlight the increased incidence and poor prognosis of lung cancer among patients with PF or IPF [9,10,11].

Notably, lung cancers arising in fibrotic lungs display distinct biological and clinical characteristics compared with those developing in non-fibrotic tissue. They frequently occur in the peripheral and lower lobes, are predominantly of the adenocarcinoma subtype with solid or sarcomatoid differentiation and often exhibit aggressive progression with limited therapeutic response. The fibrotic microenvironment enriched in TGF-β, IL-6, and characterized by increased extracellular matrix stiffness facilitates EMT, angiogenesis, and immune evasion, thereby fostering a tumor-promoting niche. Furthermore, chronic tissue injury and architectural remodeling restrict drug penetration and impair immune surveillance, suggesting that cancers developing in fibrotic lungs may follow distinct molecular trajectories and respond differently to targeted or immune-based therapies.

Accordingly, this review integrates current molecular, cellular, and translational evidence to provide a comprehensive perspective on the shared pathogenic mechanisms between PF and LC, offering insights into potential biomarkers and therapeutic targets for early detection and intervention.

## 2. Oxidative Stress, DNA Damage, and Repair

Oxidative stress, an imbalance between reactive oxygen species (ROS) production and antioxidant defenses, damages cellular components (DNA, proteins, lipids) [12]. Oxidative stress is a common pathogenic mechanism in both PF and LC, and it has been shown to activate fibroblasts, leading to excessive collagen deposition and fibrosis in PF [13]. It has been implicated in the development of lung cancer through various mechanisms, including DNA damage, activation of oncogenes, and inactivation of tumor suppressor genes [14]. Targeting oxidative stress is crucial for developing effective treatments.

PF myofibroblasts originate from diverse sources, including mesenchymal progenitor cells, resident lung fibroblasts, AECs undergoing EMT, and bone marrow-derived fibrocytes [15] and endothelial cells [16]. LC’s cellular origins are heterogeneous, including neuroendocrine cells [17] and alveolar epithelial type II cells (AECII) [18], exhibiting significant inter- and intra-tumoral variability [19]. Despite differing cellular origins, downstream consequences of inflammation and tissue remodeling share common oxidative stress-mediated pathways, creating a convergence point.

**Figure 1:**
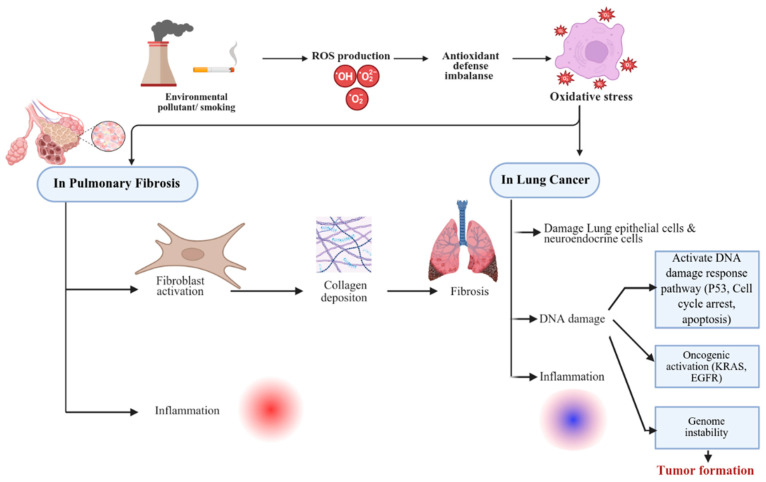
Conceptual overview of molecular mechanisms linking pulmonary fibrosis (PF) to lung cancer (LC). Chronic PF induces persistent oxidative stress, mitochondrial dysfunction, DNA damage, and remodeling of the extracellular matrix (ECM), which together create a pro-tumorigenic microenvironment. These processes may promote epithelial–mesenchymal transition (EMT), genomic instability, and chronic inflammation, facilitating LC initiation. Key signaling mediators include TGF-β, PD-1/PD-L1, and components of the tumor immune microenvironment, which contribute to immune evasion and tumor progression. References supporting each pathway are included in the main text.

Environmental pollutants and tobacco smoke, along with iron overload (through genetic mutations, disease processes, or environmental factors via the Fenton reaction) [20], generate ROS-inducing DNA damage [21], and prolonged exposure leads to the accumulation of unpaired DNA lesions. DNA repair mechanisms (e.g., DNA-dependent protein kinase, Ku protein complex) attempt to mitigate damage [22]. However, unrepaired damage activates the DNA damage response (DDR) pathway. This involves the activation of *ATM* and *ATR* kinases, triggering p53-mediated cell cycle arrest and apoptosis. It is stated that p53-mediated cell cycle arrest and DNA damage repair may exert cytoprotective effects against cancer therapies [23,24]. Phase separation dysregulation in DNA damage response can further contribute to genomic instability [25]. Breast Cancer (*BRCA1/2*) and Poly (ADP-ribose) polymerase (*PARP*) are crucial for DNA repair (homologous recombination and base excision repair), respectively [24]. Deficient repair contributes to mutations and genomic instability, promoting carcinogenesis [26]. Mitochondrial ROS (mtROS) damage mitochondrial DNA, disrupt membrane integrity, reduce mitochondrial membrane potential, impair oxidative phosphorylation, decrease ATP, and further induce DNA damage and genomic instability, increasing the risk of both carcinogenesis and fibrosis [27,28]. However, not all studies support a purely pathogenic role for oxidative stress. Controlled ROS signaling has been shown to promote epithelial regeneration and activate antioxidant defense pathways through the Nrf2 pathway, suggesting that ROS may have context-dependent protective effects during early injury repair before chronic fibrosis develops [29,30]. The intersection of oxidative stress, DNA damage, and repair in PF and LC is shown in Figure 1.

This concept of DNA damage and repair is directly relevant to the genetic alterations observed in LC and PF. PF, characterized by fibrotic and inflammatory responses, frequently involves loss-of-function mutations, e.g., *MUC5B* promoter variant rs35705950 in IPF, leading to increased *MUC5B* expression and disease exacerbation [31]. And telomere shortening (*TERT* and *TERC* mutations in familial PF) in familial PF [32]. LC, conversely, often exhibits genomic instability (chromosomal rearrangements, oncogenic gain-of-function mutation), disrupting cell cycle control and inactivating tumor suppressor gene *anaplastic lymphoma kinase* (*ALK*) rearrangements in NSCLC [33].

Clinical studies show that oxidative stress plays a major role in both lung cancer and pulmonary fibrosis. In NSCLC, elevated levels of 8-oxo-dG and malondialdehyde (*MDA*) have been consistently reported, reflecting increased oxidative DNA damage and lipid peroxidation [34]. These markers correlate with disease severity and reduced antioxidant enzyme activities, such as superoxide dismutase (*SOD*) and glutathione peroxidase (*GPx*), indicating an overwhelmed antioxidant defense system [34]. In IPF, persistent oxidative stress and mitochondrial dysfunction contribute to DNA damage and reduced antioxidant capacity [35,36]. The same imbalance between oxidative injury (8-oxo-dG, MDA) and antioxidant defense (*SOD*, *GPx*) suggests shared molecular mechanisms between fibrosis and tumor progression. Collectively, elevated 8-oxo-dG and MDA, alongside reduced *SOD* and *GPx*, serve as reliable indicators of oxidative stress and disease advancement in lung cancer and fibrosis [35,36,37]. Overall, oxidative stress connects pulmonary fibrosis and lung cancer through DNA damage, impaired repair, and genomic instability, emphasizing the need for balanced redox control in disease prevention.

## 3. Immune Responses and Microenvironment

It is stated that cancer microenvironmental cells are immune cells that exert profound effects on cancer cells [38]. Immune response regulation is crucial for the development and progression of both PF and LC. In PF, inflammation is a key driver of excessive collagen deposition in the lungs, leading to scarring and impaired lung function [39]. Damaged lung epithelial cells, neutrophils, macrophages, and other cells release inflammatory factors such as tumor necrosis factor-alpha (TNF-α), interleukin-6 (IL-6), and transforming growth factor-beta (TGF-β) [40]. These factors stimulate excessive ECM deposition and tissue stiffening by activating fibroblasts and myofibroblasts [41,42,43].

While inflammation is crucial in PF, immune dysregulation in cancer leads to immune evasion. LC development and progression are linked to immune evasion. Effective anti-tumor immunity requires T-cell infiltration, often hindered by cancer cells that inhibit T-cell receptor signaling, reduce T-cell proliferation and cytokine production, and suppress T-cell cytotoxicity through immune checkpoint molecules like *PD-1* (*encoded by PDCD1*) *and PD-L1* (*encoded by CD274*) [44]. In IPF, the chronic inflammatory microenvironment and persistent oxidative stress contribute to DNA damage, genomic instability, and activation of pro-oncogenic pathways. TGF-β signaling is a central pathway in fibrosis, inducing the transformation of fibroblasts into myofibroblasts, which are key effector cells in ECM deposition. High mitochondrial damage also leads to ER stress, both contributing to lung injury and IPF development [36]. These processes are indicative of a dysregulated cellular environment where immune responses are likely altered.

**Figure 2:**
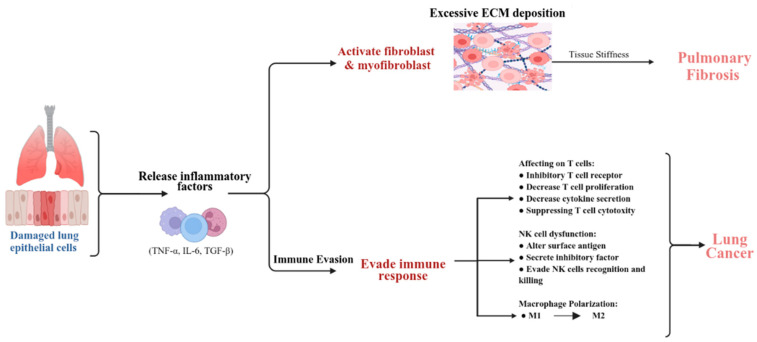
Immune response and extracellular matrix (ECM) dysregulation in pulmonary fibrosis (PF) and lung cancer (LC). This schematic illustrates the interplay between immune responses and ECM remodeling in PF and LC. In PF, injured lung epithelial cells release inflammatory mediators, including TNF-α, IL-6, and TGF-β, which activate fibroblasts and myofibroblasts, leading to excessive ECM deposition and increased tissue stiffness. In LC, immune evasion mechanisms disrupt T cell function (reduced proliferation, cytokine production, and cytotoxicity) and impair natural killer (NK) cell recognition and killing through altered surface markers and inhibitory factor secretion. Macrophage polarization is skewed toward the M2 phenotype, further promoting a pro-tumorigenic microenvironment. This figure highlights the shared mechanisms by which immune dysregulation and ECM remodeling contribute to both fibrogenesis and tumor progression.

Chimeric Antigen Receptor T-cell (CTLA-4), another critical immune checkpoint molecule, inhibits T-cell activation and proliferation by competitively binding to B7 molecules [45]. NK cells are crucial in early-stage lung cancer, as they recognize and kill cancer cells; however, cancer cells can evade this by altering surface antigens or secreting inhibitory factors. Beyond classical NK cell receptors (KIRs, LIRs, and NKG2A), numerous other immune checkpoints contribute to NK cell dysfunction in various cancers and chronic infections, including CTLA-4, PD-1, B7-H3, as well as LAG-3, TIGIT & CD96, TIM-3, checkpoint members of the Siglecs family (Siglec-7/9), CD200, and CD47 [46]. Macrophage polarization within the tumor microenvironment (TME) significantly impacts disease progression; M1 macrophages exhibit anti-tumor effects, while M2 macrophages promote tumor angiogenesis through the secretion of VEGF and other pro-angiogenic factors, thereby supporting tumor growth and tissue repair [47]. Regulatory T cells (Tregs) secrete immunosuppressive cytokines like IL-10 and TGF-β; IL-10 and TGF-β suppress anti-tumor immunity and promote tumor growth [48]. The intersection of immune response and extracellular matrix in PF and LC is shown in Figure 2.

This immunosuppressive TME poses challenges for effective cancer therapies, including the use of chimeric antigen receptor (CAR) T cells to significantly inhibit tumor growth and improve survival by targeting tumor-associated antigens like EpCAM, EGFR, and ROR1 [49,50]. EpCAM-directed CAR-T cells effectively accumulate in tumors in the brain metastasis model, reducing tumor growth without the central nervous system or systemic toxicity [51]. In PF, CAR-T cell therapy targeting fibrosis-related antigens is under investigation [52]. However, the immunosuppressive TME and antigen heterogeneity in LC complicate CAR-T cell effectiveness [53]. Combination therapies, such as pairing CAR-T cells with immune checkpoint inhibitors (e.g., anti-PD-1), are being explored to enhance antitumor effects [54]. Although PD-1/PD-L1 and TGF-β pathways are commonly linked to immune evasion, several studies demonstrate that transient activation of these signals can also mitigate acute inflammation and tissue injury, acting as compensatory immune regulators. For instance, PD-L1 upregulation may serve as a feedback mechanism to prevent excessive T-cell activation during tissue repair [55,56,57].

In PF, TGF-β, a key regulator, activates fibroblasts via the SMAD signaling pathway, inducing myofibroblast differentiation and increasing collagen production [58]. Moreover, it was stated that TGF-β could promote EMT in tumor cells and facilitate invasion and metastasis of cancer through regulation of cell survival, angiogenesis, vascular integrity, and interaction with the TME [59]. Similar to TGF-β’s role in PF, certain cytokines contribute to immune suppression in cancer, fostering tumor growth and metastasis [43].

In addition, ECM is crucial in both PF and LC as it significantly influences TME. In PF, fibroblast activation and proliferation increase collagen and fibronectin (*Fn1*) levels in the ECM, leading to excessive accumulation and increased stiffness, promoting the TGF-β/SMAD2/3 signaling pathway in a *LEMD3*-dependent manner [60]. Moreover, the altered ECM disrupts the balance of MMPs and TIMPs, and the stiffened ECM activates mechanotransduction pathways, perpetuating the fibrotic cycle [61,62,63]. In cancer, ECM components (collagen, elastin, fibronectin, and laminin) reduce treatment efficacy and are associated with poor prognosis, growth, metastasis, and drug resistance [64]. ECM changes (stiffness, degradation) significantly affect tumor growth and progression [65]. In LC, cancer-associated fibroblasts (CAFs) are crucial components of the TME; they promote tumor growth, invasion, and metastasis, exhibiting distinct metabolic profiles compared to normal fibroblasts. CAFs primarily utilize glycolysis to produce lactate, which can then fuel cancer cells, a phenomenon known as the “reverse Warburg effect”. This metabolic interplay between CAFs and cancer cells highlights another avenue for in vitro investigation into therapeutic strategies [66,67]. It was stated that high expression of fibroblast activation protein correlates with poor prognosis in high-grade serous ovarian cancer [68]. Tumor-derived type III collagen maintains tumor dormancy; its disruption reactivates tumor cell proliferation via DDR1-mediated STAT1 signaling and facilitates metastasis [69]. The stiffness of the ECM also hinders drug penetration into tumor regions [65]. ECM also modulates the behavior of immune cells by creating an immunosuppressive microenvironment, thus reducing the effectiveness of immunotherapy [69,70]. Therefore, manipulating ECM stiffness and composition to modulate the TME is a potential anti-tumor strategy [62].

## 4. Cell Activation, Proliferation, Cell Cycle, and Anti-Apoptotic Mechanisms

Cellular activation, proliferation, cell cycle regulation, and anti-apoptotic mechanisms are critical in the pathogenesis of both PF and LC. In PF, Fibroblast activation, proliferation, and fibrotic differentiation are regulated by multiple signaling pathways, including TGF-β, the Hedgehog, Wnt/β-catenin, and the Notch [71]. These pathways also influence LC; For example, Wnt pathway activation stimulates LC cell proliferation and survival [71]. In addition, LC cells utilize various mechanisms to evade apoptosis and enhance proliferation [72]. Tyrosine kinases play pivotal roles in the signaling pathways that drive lung cancer progression, particularly in NSCLC, where their dysregulation leads to uncontrolled cell growth, proliferation, differentiation, metabolism, and survival [73]. These enzymes function by phosphorylating tyrosine residues on substrate proteins, acting as molecular switches that regulate various cellular processes. Aberrant activation or overexpression of these kinases is a hallmark of cancer, providing critical targets for therapeutic interventions [74]. Several signaling pathways promote cell proliferation and tumor growth, including PI3K/Akt/mTORC1 [75], Ras/Raf/ERK [76], EGFR [77], FGFR [78], HER2 [79], and CDK4/6 [80]. A critical factor in this process is telomerase, which serves as a nearly universal biomarker for advanced human cancers [81]. Telomerase activation maintains telomere length, preventing cellular senescence and death and enabling continuous proliferation [82]. Increased telomerase activity is notably observed in NSCLC and SCLC [83]. Most tumor cells maintain telomere length through telomerase, while a minority utilize the alternative lengthening of telomere mechanism [84]. High telomerase expression correlates with increased proliferation, invasiveness, and anti-apoptotic capacity, making it a potential therapeutic target [85]. Interestingly, telomerase induction is also increased in the lung fibroblasts of IPF patients [86], while alveolar epithelial type II (AECII) in PF patients exhibit significant telomere shortening and DNA damage [87]. This is consistent with fibroblast proliferation and epithelial cell damage in PF. Furthermore, *TERT*/*TERC* gene mutations increase the risk of IPF, indicating that telomere length and maintenance capacity are important prognostic factors and potential therapeutic targets [88].

Dysregulated cell cycle control plays a crucial role in the progression of both LC and PF in LC. In AECII of IPF patients, increased *p21* expression induces cell cycle arrest and senescence, inhibiting cell differentiation. *p21* overexpression promotes myofibroblast activation via profibrotic cytokine release, contributing to fibrosis [89]. Regulator of cell cycle (*RGCC*) overexpression delays early Smad2/3 phosphorylation induced by TGF-β, having a protective effect in PF [90]. In NSCLC and SCLC, abnormal expression of cell cycle genes (*TP53*, *p21*, and *cyclin D1*) is associated with high proliferative and invasive tumors [91]. The long noncoding RNA *HOX Transcript Antisense RNA* (*HOTAIR*) is a marker of cell cycle abnormalities, promoting G1-S phase transition via Rb-E2F pathway regulation and accelerating cell proliferation and invasion via EMT and β-catenin pathways [92]. Small molecule cell cycle regulators such as WEE1 [93] and aurora kinase B inhibitors [94] show potential in SCLC treatment.

Dysregulation of cell death pathways, including apoptosis, contributes to both PF and LC progression. In PF, the *PARP-1* inhibitor HYDAMTIQ reduces fibrosis and inflammation in preclinical models by blocking the TGF-β/Smad pathway and reducing fibroblast activation [95]. Cancer pathogenesis also involves dysregulation of cell death pathways, mirroring the situation in PF. This dysregulation includes imbalances in Bcl-2 family proteins, various inhibitors of apoptosis proteins (IAPs), dysregulation of repair mechanisms, activation of *P53*, and death receptors [64]. The therapeutic exploitation of these dysregulations is evident in the use of PARP inhibitors like Olaparib and Niraparib. These drugs trap PARP-DNA complexes, hindering repair and inducing apoptosis, thereby enhancing cancer treatment efficacy [96]. Survivin overexpression promotes cancer progression and therapy resistance [97]. Targeting anti-apoptotic mechanisms represents a promising therapeutic avenue for both diseases [97]. Conversely, some studies highlight the dual nature of these signaling pathways. For instance, moderate activation of PI3K/Akt and Wnt/β-catenin can promote epithelial repair and protect against apoptosis during acute lung injury, suggesting that complete inhibition may impair regeneration [98]. In summary, immune dysregulation and extracellular matrix remodeling create a shared pathogenic microenvironment in pulmonary fibrosis and lung cancer, fostering chronic inflammation, immune evasion, and tumor progression.

## 5. Mitochondrial Metabolism

Mitochondrial dysfunction, often a consequence of oxidative stress, is a crucial factor in both PF and LC [99]. Mitochondria are a major source of ROS, particularly during oxidative phosphorylation. Mitochondrial dysfunction often leads to increased ROS production, contributing to oxidative stress and lipid peroxidation, both key drivers of ferroptosis [100]. Moreover, mitochondrial DAMPs, particularly mtDNA and cardiolipin, released as a result of mitochondrial injury in both IPF and lung cancer, can activate innate immune pathways such as TLR9 and cGAS–STING, initiating proinflammatory signaling that further amplifies tissue injury and tumor-promoting inflammation [101].

Ferroptosis, an iron-dependent form of cell death driven by lipid peroxidation and ROS accumulation and closely linked to mitochondrial function, is emerging as a promising therapeutic target in both LC and PF [102,103]. In LC, inducing ferroptosis inhibits cell proliferation and growth [102]. Ferroptosis regulators, such as glutathione peroxidase 4 (GPX4), can promote chemoresistance by suppressing ferroptosis. Conversely, GPX4 inhibitors enhance the efficacy of platinum-based anti-cancer drugs [104]. In PF, increased iron deposition and ferroptosis are observed in IPF lung tissue. Iron exacerbates fibrosis by inducing AECII death and mitochondrial dysfunction [103] and ferroptosis accelerates PF pathology by promoting EMT and ECM accumulation [105].

**Figure 3:**
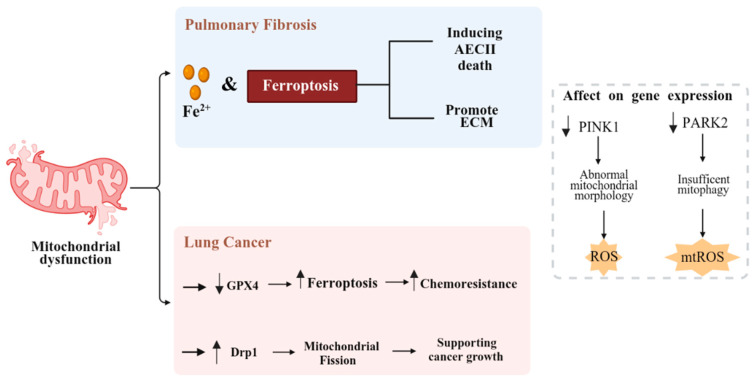
The role of mitochondrial metabolism in pulmonary fibrosis (PF) and lung cancer (LC). This schematic depicts the contribution of mitochondrial metabolism to PF and LC pathogenesis. In PF, iron accumulation (Fe^2+^) and ferroptosis drive alveolar epithelial type II (AECII) cell death, promoting extracellular matrix (ECM) deposition and tissue fibrosis. In LC, mitochondrial dysfunction is associated with decreased GPX4 expression, enhancing ferroptosis resistance and chemoresistance, while increased Drp1 expression promotes mitochondrial fission to support tumor growth. Dysregulated expression of PINK1 and PARK2 in both conditions contributes to abnormal mitochondrial morphology, impaired mitophagy, and elevated reactive oxygen species (ROS and mtROS) production. This figure highlights how mitochondrial dysregulation links cellular stress, ferroptosis, and disease progression in PF and LC.

Mitochondrial homeostasis significantly impacts cellular function in both LC and PF. In LC, mitochondrial dynamics, regulated by proteins like dynamin-related protein 1 (Drp1) that promote mitochondrial fission, influence cell proliferation, invasion, and metastasis [106]. Mitophagy, the selective removal of damaged mitochondria, is essential for mitochondrial quality control. PTEN-Induced Kinase 1 (PINK1) and Parkin proteins mediate the autophagic degradation of damaged mitochondria, maintaining cellular energy metabolism and functional stability and homeostasis [107]. In LC, mitophagy’s role varies under different stress conditions; methyltransferase-like 3 (METTL3) promotes cisplatin and etoposide chemoresistance in SCLC by upregulating PINK1-Parkin-mediated mitophagy [108]. In PF, reduced *PINK1* expression in AECII leads to abnormal mitochondrial morphology and increased ROS generation; Decreased *PARK2* expression in IPF myofibroblasts results in insufficient mitophagy, leading to mtROS generation and Platelet-derived growth factor receptor (PDGFR)/mTOR signaling activation, and increased myofibroblast differentiation and proliferation [109,110]. Nevertheless, emerging evidence indicates that mitophagy can have both profibrotic and antifibrotic outcomes, depending on cellular context. Excessive mitophagy may deplete functional mitochondria and impair energy metabolism, while insufficient mitophagy promotes oxidative damage [111]. So, mitochondrial dysfunction and ferroptosis are key shared mechanisms in pulmonary fibrosis and lung cancer, linking oxidative stress, iron overload, and impaired mitophagy to fibrosis progression, tumor growth, and therapy resistance. The function of mitochondrial metabolism in PF and LC is shown in Figure 3.

## 6. The Ubiquitin–Proteasome System in LC and PF

The ubiquitin–proteasome system (UPS) plays a critical role in protein degradation and influences various cellular processes [112]. The UPS plays a crucial role in cancer development, with an imbalance between ubiquitin E3 ligases and deubiquitinases frequently contributing to tumorigenesis and therapeutic resistance [113].

In NSCLC, abnormal ubiquitin modifications are implicated in oncogenesis [114]. High expression of ubiquitin-specific protease USP39 is associated with increased proliferation and drug resistance in LC cells [115]. The 26S proteasome promotes tumorigenesis by regulating the stability and function of tumor suppressor proteins such as p53 [116]. Proteasome inhibitors, such as MG132, induce apoptosis in lung cancer cells by increasing oxidative stress and depleting glutathione, enhancing chemotherapy effectiveness and reducing drug resistance [117].

In PF, 26S proteasome activity is significantly increased, particularly during TGF-β-induced myofibroblast differentiation, with Rpn6 subunit regulation playing a key role [118]. The UPS significantly impacts PF pathogenesis by regulating EMT and intracellular protein homeostasis [16]. The FDA-approved proteasome inhibitor Bortezomib inhibits *TGF-β1*-induced gene expression in fibroblasts by preventing Smad-mediated transcription and collagen deposition, and it inhibits BLM-induced fibrosis in mice lungs [119]. However, the less toxic, more selective proteasome inhibitor augmented BLM-induced PF in a mouse model following local lung application, highlighting the need for further investigation into the safety and efficacy of proteasome inhibitors in clinical [119]. Interestingly, proteasome inhibition does not uniformly suppress fibrosis or cancer progression. Certain studies indicate that partial proteasome inhibition may disrupt cellular homeostasis and exacerbate stress responses, particularly in lung tissue with high metabolic demand [120].

The UPS represents a promising therapeutic target for both LC and PF. Modulating UPS activity, either through proteasome inhibition or targeting specific deubiquitinases, could offer novel therapeutic strategies. However, the complex roles of the UPS necessitate careful consideration of potential off-target effects and the development of highly specific inhibitors to maximize therapeutic benefit and minimize adverse effects.

## 7. Biomarkers in Lung Fibrosis and Lung Cancer

Given the shared molecular landscape between pulmonary fibrosis and lung cancer, several classes of biomarkers are being actively investigated for early cancer detection in individuals with fibrotic lungs. Genetic and epigenetic biomarkers play a pivotal role in this regard. Bioinformatics analyses have identified numerous differentially expressed genes (DEGs) and hub genes in IPF that are also implicated in lung cancer [74]. For instance, a study reported 1888 DEGs in IPF—comprising 1105 upregulated and 783 downregulated genes—and identified 10 hub genes potentially crucial for the pathogenesis of IPF and its association with lung cancer [74]. These genes could serve as valuable indicators of increased cancer risk in patients with fibrotic lungs. RNA modifications, particularly N6-methyladenosine (m6A), have also emerged as promising epigenetic biomarkers for both lung cancer and pulmonary fibrosis, suggesting potential utility in identifying early cancer progression within fibrotic lungs [121].

Noncoding RNAs (ncRNAs) have drawn significant attention as diagnostic tools. Exosomal miRNAs are highly stable and easily measurable in biofluids, making them attractive non-invasive biomarkers. Dysregulated miRNA expression has been observed in various lung diseases, including silicosis-related lung fibrosis and NSCLC [122]. The detection of specific miRNA expression profiles in fibrotic individuals could thus provide early indications of malignant transformation. Likewise, circRNAs, which are also found in exosomes, have demonstrated potential as biomarkers for lung cancer due to their high stability and involvement in tumor progression and metastasis [121]. Monitoring the levels of particular circRNAs in fibrotic patients might help identify the onset of cancer development.

Protein biomarkers represent another major class of diagnostic targets. Extracellular heat shock proteins (eHSPs) are currently being investigated as biomarkers and therapeutic targets in fibrosing interstitial lung diseases (ILDs) [123], as their altered expression may indicate a shift toward malignant transformation. Furthermore, oncogenic and immune checkpoint proteins, such as EGFR, ALK, KRAS G12C, and PD-L1, which are well-established in lung cancer, could be monitored in fibrotic patients to detect early malignancy or to guide preventive therapies [124]. A broad range of cancer-associated proteins, including HER2, c-MET, MUC1, EGFR, and PD-L1, are also expressed in lung cancer [125]. Tracking their alterations in the context of lung fibrosis could facilitate early diagnosis and intervention.

The lung microbiome has also emerged as a potential source of diagnostic biomarkers. Alterations in the composition and function of the lung microbiota are increasingly linked to lung cancer development [121]. Analyzing circulating microbial DNA from sources such as bronchoalveolar lavage fluid (BALF), thoracentesis samples, or liquid biopsies could serve as a non-invasive means of detecting early cancerous changes in fibrotic lungs [121].

Together with these molecular and microbial indicators, many of the key mediators involved in fibrotic remodeling also participate in carcinogenic progression, reinforcing their value as dual-purpose biomarkers. Several of these molecules—such as TGF-β, IL-6, VEGF, and MMPs—are summarized in the Supplementary Table S1 and exemplify how inflammatory signaling, extracellular matrix remodeling, and cellular transitions like EMT intersect in both fibrosis and malignant transformation. These shared mechanisms are strengthened by common pathways, including PI3K/AKT and Wnt/β-catenin, which not only drive fibrotic progression but also facilitate tumor invasion and metastasis. Collectively, this convergence highlights promising therapeutic opportunities, with biomarkers such as CXCL12, MMPs, and EMT-related factors offering avenues to disrupt processes that contribute to both pulmonary fibrosis and lung cancer (Table S1).

## 8. Clinical Implications and Future Directions

Integrating advanced genomic and proteomic technologies, such as large-panel next-generation sequencing (NGS) in conjunction with Molecular Tumor Boards (MTB), is vital for identifying comprehensive molecular profiles that guide precision medicine, particularly in fibrotic lungs with cancer risk [126]. This personalized approach facilitates the discovery of both established and emerging biomarkers, enabling more tailored and effective treatment strategies [126]. The development of non-invasive biomarkers from exosomes in biofluids offers additional promise for early disease detection and longitudinal monitoring, particularly for fibrotic lung conditions such as silicosis [127]. Meanwhile, artificial intelligence (AI) and machine learning are being employed to analyze complex biological datasets, uncovering novel biomarkers and therapeutic targets while addressing the interpretability challenges inherent to these models [126]. Future investigations will likely leverage transcriptomic and systems biology approaches to unravel the mechanisms underlying lung diseases and to identify new therapeutic avenues—especially for conditions like ischemia–reperfusion injury in lung transplantation [126]. The overarching goal is to achieve early prediction, accurate prognosis, and effective intervention strategies that improve patient outcomes by targeting the shared and distinct molecular pathways underlying lung fibrosis and lung cancer.

**Figure 4:**
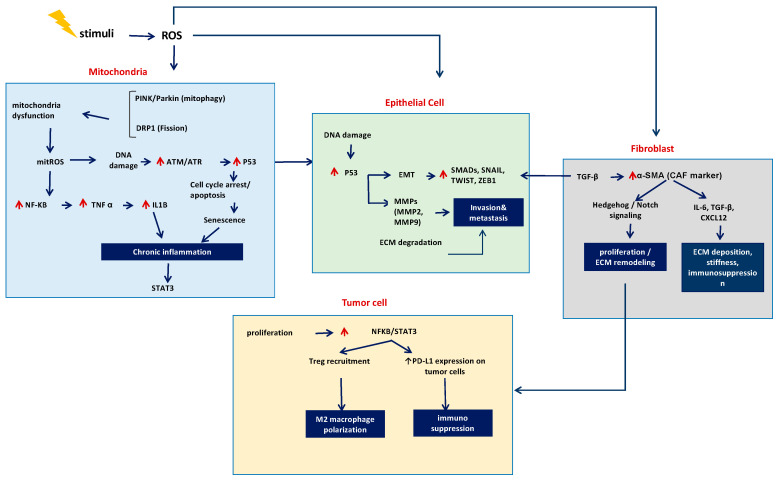
Integrated Pathogenic Pathways Linking Mitochondrial Dysfunction, Epithelial Damage, Fibroblast Activation, and Tumor Progression in the Tumor Microenvironment. Mitochondrial stress increases mitoROS, triggering ATM/ATR–p53 signaling, cell-cycle arrest, senescence, and NF-κB-mediated chronic inflammation. In epithelial cells, DNA damage and p53 activation enhance EMT transcription factors (SMADs, SNAIL, TWIST, ZEB1), MMP production, and ECM degradation, driving invasion and metastasis. TGF-β-induced fibroblast activation generates α-SMA^+^ CAFs, which promote ECM remodeling and immunosuppressive cytokine release (IL-6, TGF-β, CXCL12). Tumor cells amplify NF-κB/STAT3 signaling, increase PD-L1 expression, recruit Tregs, and induce M2 macrophage polarization, collectively fostering an immunosuppressive, pro-tumorigenic microenvironment.

The close crosstalk between the development of PF and LC highlights the shared pathogenic mechanisms and key signaling molecules involved. It summarizes how oxidative and mitochondrial stress trigger DNA damage, genomic instability, and altered cell cycle control, leading to fibroblast activation and ECM accumulation in PF and enhanced proliferation and metastasis in LC. Cytokines such as TGF-β, IL-6, TNF-α, and IL-10, along with signaling cascades like PI3K/Akt/mTOR, Wnt/β-catenin, Hippo/YAP, and Hedgehog/Notch, mediate inflammation, EMT, and immune dysregulation. Key regulators, including *GPX4*, *HOTAIR*, *USP15*, *USP39*, *TP53* (*p53*), *and* *CDKN1A* (*p21*), further connect ferroptosis, apoptosis, and metabolism. Together, these interactions highlight the molecular convergence of PF and LC and reveal potential shared therapeutic targets (Figure 4).

## 9. Conclusions

PF and LC, while clinically distinct, exhibit a complex interplay at the molecular level, driven by shared mechanisms that create a microenvironment conducive to malignant transformation. This review delineated these convergent pathways, emphasizing the roles of oxidative stress, DNA damage and repair, immune microenvironment remodeling, mitochondrial metabolism, and the ubiquitin–proteasome system in the pathogenesis of both diseases. We highlighted how persistent inflammation, genomic instability, and aberrant signaling pathways, such as TGF-β, *Wnt*, and PI3K/Akt/*mTOR*, contribute to fibrogenesis and tumorigenesis alike. Furthermore, we explored the impact of ECM remodeling, immune evasion, and altered cellular metabolism on disease progression and therapeutic resistance.

## Supplementary Materials

The following supporting information can be downloaded at https://www.mdpi.com/article/10.3390/cancers17233861/s1. Table S1: Key Molecular Mediators Linking Pulmonary Fibrosis and Lung Cancer [128,129,130,131,132,133,134,135,136,137,138,139,140,141].
