# Supplementary material for: From Fibrosis to Malignancy: Mechanistic Intersections Driving Lung Cancer Progression
Source: Cancers (Basel). 2025 Dec 1;17(23):3861. doi: 10.3390/cancers17233861 (PMC12691483; doi:10.3390/cancers17233861)
Supplement: Supplementary file 1 [file cancers-17-03861-s001.zip › cancers-3953751-supplementary.pdf]

cancers

MDPI

Supplementary table

Table S1. Key Molecular Mediators Linking Pulmonary Fibrosis and Lung Cancer.

Category	Molecule/Marker	Role in PF	Role in LC	Shared Pathways/Impact	Ref.
**Growth Factors**	TGF-β	Promotes fibrosis by activating EMT and inducing fibroblast differentiation.	Drives EMT, tumor growth, and metastasis.	**EMT**: Induces epithelial to mesenchymal transition, important in fibrosis and cancer progression.	[128,129]
**Cytokines**	IL-6	Mediates inflammatory signaling, contributes to fibroblast activation.	Facilitates tumor proliferation, angiogenesis, and metastasis by activating the STAT3 pathway.	**Chronic Inflammation**: Sustains inflammatory microenvironment characteristic of both diseases.	[130–132]
**Chemokines**	CXCL12	Stimulates fibroblast migration and enhances extracellular matrix deposition.	Promotes tumor cell invasion and metastasis via binding to CXCR4.	**Fibroblast Recruitment and Tumor Invasion**: Shared signaling promotes EMT and stromal remodeling.	[133,134]
**Matrix Metalloproteinases (MMPs)**	MMP1, MMP9, MMP13	Induce extracellular matrix degradation and remodeling, enabling fibrotic progression.	Support cancer cell invasion by breaking basement membranes and extracellular matrix.	**Matrix Remodeling**: These enzymes mediate structural deformation during both fibrosis and cancer metastasis.	[135–137]
**Fibroblast Activation Markers**	α-SMA, CTGF	Indicators of myofibroblast differentiation; promote ECM deposition.	Facilitate cancer-associated fibroblast (CAF) transformation, supporting tumor growth.	**Fibroblast Phenotypes**: Activated fibroblasts/myofibroblasts are essential in stromal support and matrix production.	[138,139]
**Angiogenesis Factors**	VEGF	Encourages vascular proliferation in fibrotic tissue, exacerbating hypoxia.	Enhances tumor angiogenesis, creating microenvironments favorable for cancer progression.	**Angiogenesis**: Shared vascular remodeling process contributes to pathological tissue growth.	[140]

**EMT Markers**	Snail, Twist1, E-cadherin, N-cadherin	Promote transition of epithelial cells to myofibroblasts.	Drive EMT-associated metastasis by enabling cellular motility and invasive traits.	**Cellular Plasticity**: EMT drives fibrosis and cancer invasion through cyclical epithelial and mesenchymal states.	[134,141]
**Non-Coding RNAs**	LncRNA (e.g., H19, LINC01133)	Regulate fibroblast activation and ECM production via signaling pathways.	Modulate gene expression and metabolic pathways to drive tumor cell invasiveness.	**Transcriptomic Regulation**: Non-coding RNAs influence gene expression networks central to tissue remodeling and oncogenesis.	[134,139]
**Lipids and Lipid Mediators**	Sphingolipids	Contribute to fibroblast signal modulation and ECM composition.	Facilitate cancer cell survival and proliferation through metabolic adaptation.	**Membrane Dynamics**: Enhance stability and signaling crucial for both fibrotic and tumor-derived cell survival.	[136,137]
